# Damage of Neuroblastoma Cell SH-SY5Y Mediated by MPP^+^ Inhibits Proliferation of T-Cell Leukemia Jurkat by Co-Culture System

**DOI:** 10.3390/ijms150610738

**Published:** 2014-06-13

**Authors:** Fuli Wang, Umer Farooq Awan, Yuanyuan Wang, Luna Wang, Hong Qing, Hong Ma, Yulin Deng

**Affiliations:** School of Life Science, Beijing Institute of Technology, No. 5 Zhongguancun Nandajie, Haidian District, Beijing 100081, China; E-Mails: wangfuli5728@163.com (F.W.); umeraliawan@gmail.com (U.F.A.); mah_cindy@sina.com (Y.W.); wangluna1955@gmail.com (L.W.); hqing@bit.edu.cn (H.Q.)

**Keywords:** Parkinson’s disease, co-culture, human T-cell leukemia, cell cycle, necrosis

## Abstract

The adaptive immune system has implications in pathology of Parkinson’s disease (PD). Research data demonstrated that the peripheral CD4^+^ T-cell population decreased in pathogenesis of PD. The effect of damaged dopaminergic neurons on peripheral T cells of PD is still unknown. In this study, we constructed a neuronal and glial cells co-culture model by using human neuroblastoma cells SH-SY5Y and gliomas cells U87. After the co-culture cells were treated with neurotoxin 1-methyl-4-phenylpyridinium (MPP^+^) for 24 h, the conditioned media was harvested and used to cultivate T-cell leukemia Jurkat cells for another 24 h. We then analyzed the cell proliferation, cell cycle and necrosis effect of Jurkat cells. The results showed that co-culture medium of SH-SY5Y and U87 cells with MPP^+^ treatment inhibited the proliferation of Jurkat cells compared to control medium without MPP^+^, even though the same concentration of MPP^+^ had very little toxicity to the Jurkat cell. Furthermore, co-culture medium with low concentration of MPP^+^ (100 µM) arrested Jurkat cells cycle in G2/M phase through increasing cell cycle division 2 (CDC2) and CyclinB1 expression level, whereas co-culture medium with high concentration of MPP^+^ (500 µM) induced Jurkat cell necrosis through cellular swelling and membrane breakage. Our data implies that damaged dopamine neurons with glial cells can lead to the reduced number or inhibited proliferation activity of peripheral T cells.

## 1. Introduction

Parkinson’s disease (PD) is the second most common neurodegenerative disease characterized by progressive loss of dopamine neurons within the substantianigra pars compacta (SNpc) and a related shortage of neurotransmitter dopamine in nigrostriatum [1,2]. To understand the mechanism of neuronal cell death in PD, numerous studies have been carried out in postmortem brains, as well as experimental cell and animal models of PD [3,4]. MPTP (1-methyl-4-phenyl-1,2,3,6-tetrahydropyridine) is one of the most widely used neurotoxins which could induce dopamine neuron death and Parkinson’s disease in mice and primates. MPP^+^ (1-methyl-4-phenyl-2,3-dihydropyridiumion) is the product of MPTP, which is converted from MPTP by monoamine oxidase-B in the astrocyte [5]. It is subsequently taken up by dopamine (DA) neurons through dopamine transporters (DAT) and interacts with the mitochondrial respiratory chain and damages the mitochondrial complex-1, thereby increasing the level of oxidative stress and leading to cell death [6].

Previous studies indicated that an adaptive immune system was important in the pathology of PD, and CD4^+^/CD8^+^ T cells appeared in postmortem brain samples from PD patients [7,8,9,10]. Moreover, infiltrated T cells in brain could induce the blood vessel change during PD progression [9,11], and the peripheral T-cell pool also can be changed in PD [12,13]; the CD4^+^ population especially decreased [14]. The reasons for T-cell changes are not yet known. Jurkat is the human CD4^+^ T cell leukemia cell line which has been thoroughly characterized and widely used as a stable cell model to study T-cell function and signal pathway [15,16].

At present, neuron and glial cell co-culture techniques have been well established to mimic the conditions in the brain, whereby neurons are surrounded by glial cells. Using this co-culture cell model, we cannot only investigate neuron–glial interaction in Parkinson’s disease [17], but also study the effect of damaged neurons on the peripheral T cell. Thus, in this study, we treated co-cultures of human neuroblatoma SH-SY5Y and human U87 glioblastoma cells with two different amounts of MPP^+^, and then we analyzed the effects of the use of the conditioned media to cultivate Jurkat cells. Our results indicated that damaged neuroblastoma cells SH-SY5Y inhibited proliferation of Jurkat cell, and SH-SY5Y cells damaged by 100 µM neurotoxin MPP^+^ could induce the G2/M cell-cycle checkpoint for Jurkat cells, resulting in necrotic Jurkat cells death when increasing the concentration of MPP^+^ to 500 µM.

## 2. Results and Discussion

### 2.1. Cell Viability after MPP^+^ Treatment

**Figure 1:**
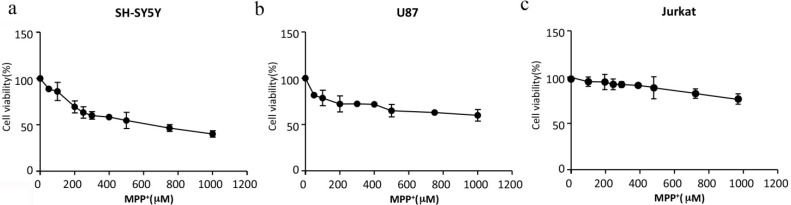
MPP^+^ had the most toxicity on SH-SY5Y cells. (**a**) Cellular viability of SH-SY5Y cells treating with MPP^+^; (**b**) Cellular viability of U87 cells treating with MPP^+^; (**c**) Cellular viability of Jurkat cells treating with MPP^+^. Data presented are mean ± SEM values of at least three independent experiments.

Firstly, a control experiment was done to evaluate the cell proliferation of Jurkat and U87 cells in the medium of SH-SY5Y cells (Dulbecco Modified Eagle Medium (DMEM)). There was no obvious difference from their original medium (1640 medium for Jurkat cells, MEM medium for U87 cells) (data not shown). Thus, the DMEM medium was added in the followed co-culture system. The second control experiment was done to analyze the effects of MPP^+^ on single cell cultures. We applied different concentrations of MPP^+^ ranging from 50 to 1000 μM to different cells including SH-SY5Y, U87 and Jurkat cells for 24 h, and cellular viability was measured by 3-(4,5-dimethylthiazol-2-yl)-2,5-diphenyltetrazolium bromide (MTT) (SH-SY5Y, U87) or 3-(4,5-dimethylthiazol-2-yl)-5-(3-carboxymethoxyphenyl)-2-(4-sulfophenyl)-2*H*-tetrazolium (MTS) (Jurkat) assay. It is clear that Jurkat cells are poorly affected by MPP^+^ at each concentration used and that SH-SY5Y cells are the most affected, because100 μM MPP^+^ damaged 19.9% SH-SY5Y cells but only 0.2% for Jurkat cells in 24 h, and 500 μM MPP^+^ reached semi-lethal levels of SH-SY5Y cells after 24 h, while other cells, U87 and Jurkat, still kept high survival rate (about 71.1% and 93.4%, respectively) at this dose (Figure 1). In the following study, we chose 100 μM MPP^+^ as a resistant dose (cell viability kept about 80% and 98% in SH-SY5Y cells and Jurkat cells, respectively) and 500 μM MPP^+^ as an effective dose, while this dose only damaged neuroblastoma cells SH-SY5Y (about 50% cell viability rate) but not T-cell Jurkat (about 92% cell viability rate), to evaluate the effect of slightly or semi-damaged SH-SY5Ycells on Jurkat cells.

### 2.2. Proliferation of Jurkat Cells Was Inhibited in MPP^+^-Treated SH-SY5Y and U87 Cells Co-Culture Medium

**Figure 2:**
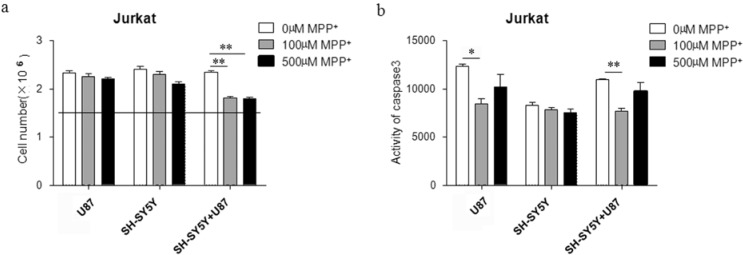
MPP^+^-treated co-culture medium of SH-SY5Y and U87 cells can inhibit proliferation of Jurkat cells dissociated with apotosis. (**a**) The cell number of Jurkat was decreased in MPP^+^-treated co-culture medium compared to control without MPP^+^ (straight line means seeding cell number). The medium derived from the incubation of SH-SY5Y or U87 cells with (or without) the presence of MPP^+^ as background control; ** *p* < 0.01 compared with Jurkat in co-culture medium without MPP^+^; (**b**) Caspase3 activity of Jurkat cells in MPP^+^-treated co-culture medium was lower than control without MPP^+^. The medium derived from the incubation of SH-SY5Y or U87 cells with (or without) the presence of MPP^+^ as background control. * *p* < 0.05 compared with Jurkat in U87 incubation medium without MPP^+^; ** *p* < 0.01 compared with Jurkat in co-culture medium without MPP^+^.

We hypothesized that the co-culture medium of MPP^+^ treating SH-SY5Y and U87 cells might inhibit proliferation of Jurkat cells. SH-SY5Y and U87 cells (seeded in a ratio of 1:1) were treated with 100 or 500 µM MPP^+^ for 24 h after 8 h of adhering, and then the co-cultured medium was reused to culture Jurkat cells for another 24 h. We subsequently analyzed the cell proliferation by checking the number of live Jurkat cells. Firstly, the medium derived from the incubation of U87 cells with (or without) the presence of MPP^+^ was used to treat Jurkat cells; we did not observe any significant difference in the live cell number (histograms on the left of Figure 2a). In a parallel experiment, the medium derived from the incubation of SH-SY5Y cells with (or without) the presence of MPP^+^ was used to treat Jurkat cells without observing any difference in the number of live cells (histograms in the middle of Figure 2a). Only when MPP^+^ both at 100 and 500 µM conditions was added to a SH-SY5Y/U87 co-culture and the conditioned medium was used to treat Jurkat cells was a significant difference in live cell number was observed (histograms on the right of Figure 2a). Although the cell toxicity (~30%, Figure 1b) of U87 cells can be observed at the dosage of 500 µM MPP^+^, the medium derived from the incubation of U87 cells cannot mediate any difference in the number of treated Jurkat cells. Our data indicated that the co-culture system releasing some sort of MPP^+^ derivate might induce inhibition effects on Jurkat cells. Moreover, a study on apoptosis was performed to further elucidate mechanism of inhibiting proliferation. The caspase3 activity of Jurkat cells was tested by Promega caspase3/7 kit, and we found it was decreased significantly after applying 100 µM of MPP^+^ and unaffected in the presence of 500 µM of MPP^+^ (Figure 2b), which demonstrated that MPP^+^ treating SH-SY5Y/U87 cells could inhibit proliferation of Jurkat cells without apoptosis induction. Moreover, in order to estimate the specificity of U87 glial cell in the co-culture system, we gave analysis of the effect of another kind of human glioma cell U251 (data not shown). Similar results indicated that only conditioned medium derived from neuron–glial cells co-culture damaged by MPP^+^ could mediate the inhibition of T-cell proliferation.

We also noticed that the caspase 3 activity measured in the presence of three different conditioned media without MPP^+^ was different (Figure 2b). SH-SY5Y medium inhibited caspase 3 activity significantly when compared to the U87 and SH-SY5Y/U87 co-culture system, but the live cell numbers of Jurkat in three kinds of these media were the same (Figure 2a). Moreover, it was shown that lower concentration of MPP^+^ had an effect of inhibiting the caspase activity in U87 cells and the SH-SY5Y/U87 co-culture system, but higher concentration had no variable effects in different media (Figure 2b). All the results indicated that the anti-apoptosis effect of Jurkat cells would be induced when the culture conditions were not so good. However, this kind of protect function was not enough to increase the cell proliferation, or because the change of live cell numbers was not dependent on the cell apoptosis.

### 2.3. Low Concentration of MPP^+^-Treated SH-SY5Y and U87 Cells Co-Culture Medium-Induced Accumulation of G2/M Phase in Jurkat Cells

**Figure 3:**
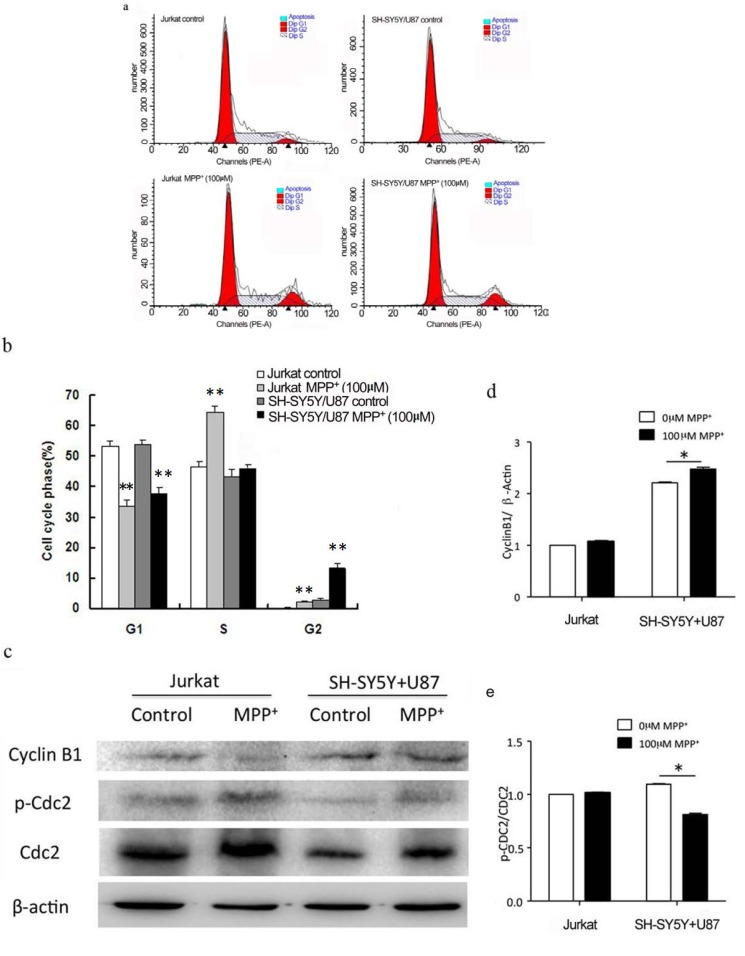
One hundred micro mole per liter MPP^+^-treated co-culture medium of SH-SY5Y and U87 cells induced Jurkat cell G2/M cell-cycle checkpoint. Jurkat cells treated with or without MPP^+^ as background control, Jurkat cells incubated with the conditioned media of co-culture system treating without MPP^+^ as control. (**a**) Cell cycle of G2/M phase of Jurkat cells was increased after 100 µM MPP^+^ application compared to control medium without MPP^+^. ▲ means the position of Dip G1 and Dip G2; Dip G1 is the left red peak and Dip G2 is the right red peak; (**b**) Statistical analysis for the effect of conditioned media after 100 µM MPP^+^ application on inducing cell cycle arrested. ** *p* < 0.01, compared with Jurkat cells in co-culture medium without MPP^+^ applied; (**c**) Western blot assay for the G2/M cell-cycle checkpoint-related proteins. Phosphorylation of CDC2 was increased while Cyclin B1 decreased in protein level of Jurkat cells in MPP^+^-treated co-culture medium; (**d**) Quantification of intensities of CyclinB1/β-actin by Bio-Rad imaging-lad 4.0 software (Bio-Rad, Richmond, CA, USA). * *p* < 0.05 compared with Jurkat cells in co-culture medium without MPP^+^ applied; (**e**) Quantification of intensities of p-CDC2/CDC2 by Bio-Rad imaging-lad 4.0 software. * *p* < 0.05 compared with Jurkat cells in co-culture medium without MPP^+^ applied.

Since Jurkat cell number decreased independent of caspase3 activation, we examined the Jurkat cell cycle by PI (Propidium Iodide) staining flow cytometry, and found that 100 µM MPP^+^-applied co-culture media made 13.15% ± 1.47% Jurkat cells in the G2/M phase compared to co-culture medium without MPP^+^ (*p* < 0.01) (Figure 3a,b), while there was no difference between the co-culture medium with and without 500 µM MPP^+^ (data not shown). Moreover, we investigated the level of CDC2 and CyclinB1 proteins (marker proteins of G2/M checkpoint) of Jurkat cells by Western blot. The phosphorylation of CDC2 decreased while the CyclinB1 protein increased in Jurkat cells in 100 µM MPP^+^ treated co-culture medium (Figure 3c–e). These results indicated an increased cell in G2/M phase might be due to down-regulation of p-CDC2 while independent of CyclinB1.

### 2.4. High Concentration of MPP^+^-Treated SH-SY5Y and U87 Cells Co-Culture Medium-Induced Jurkat Necrosis

**Figure 4:**
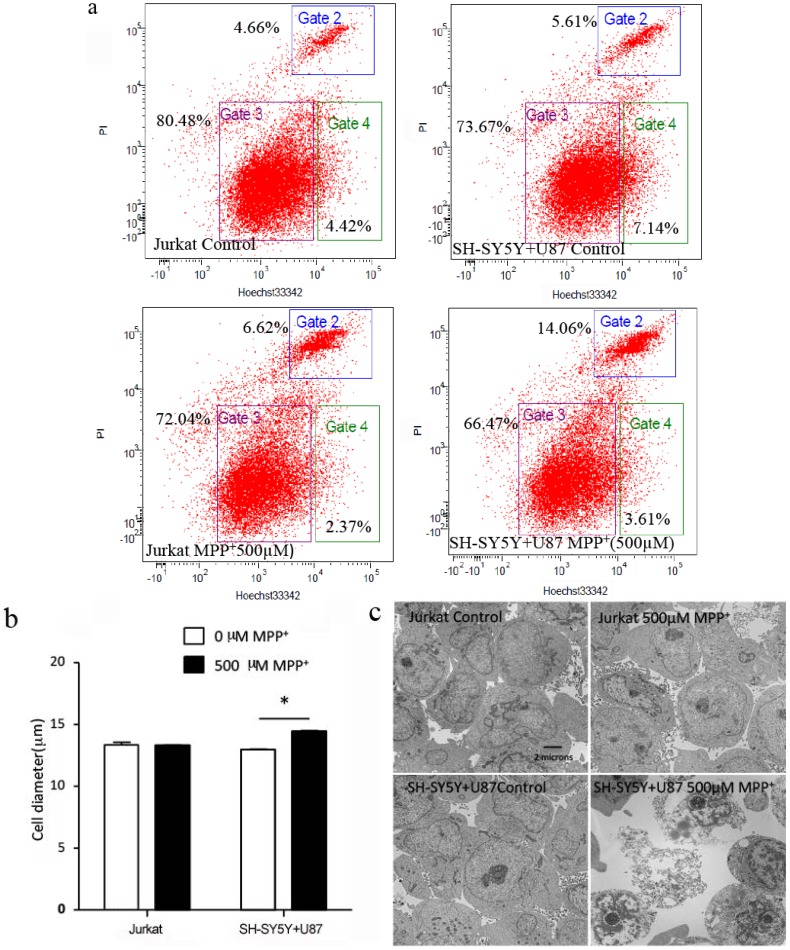
Five hundred micro mole per liter MPP^+^-treated co-culture medium of SH-SY5Y and U87 cells induced Jurkat cells necrosis. Jurkat cells treated with or without MPP^+^ as background control; Jurkat cells incubated with the conditioned media of co-culture system treating without MPP^+^ as control. (**a**) Fluorescence activating cell sort (FACS) assay for necrosis of Jurkat cells in MPP^+^-treated co-culture medium. Left channel showed Jurkat cells treated with or without MPP^+^; right channel showed Jurkat cells incubated with the conditioned media of co-culture system treating with or without MPP^+^. Cells in Gate 2 represent for necrosis, Gate 4 for apoptosis and Gate 3 for live cells. PI, Propidium iodide; (**b**) The changes of Jurkat cell diameter in MPP^+^-treated co-culture medium. * *p* < 0.05 compared with Jurkat cells in co-culture medium without MPP^+^; (**c**) Electro microscopy assay for Jurkat cell membrane damage in MPP^+^-treated co-culture medium.

As our data showed, 500 µM MPP^+^ applied co-culture medium inhibiting proliferation of Jurkat cells dissociated with caspase3 activation and cell-cycle checkpoint; we estimated the necrosis for Jurkat cells by flow cytometry analysis after Hochest33342 and PI double staining. There was 9.43% ± 1.39% increasing of Jurkat cells necrosis in 500 µM MPP^+^-treated co-culture medium compared to co-culture medium without MPP^+^ (*p* < 0.01) (Figure 4a). Necrosis may cause cell swelling and induce cell membrane breakage, and then we examined the diameter of Jurkat cells by MILLPORE Scepter (Handheld Automated Cell Counter, Millipore, Billerica, MA, USA) and cell membrane integrity by electron microscope. We found 500 µM MPP^+^-applied co-culture medium increased the diameter of Jurkat cells (Figure 4b), and transmission electron microscopic examination revealed the presence of micromorphological alterations of broken membranes (Figure 4c) compared to the control Jurkat cells incubated with the conditioned co-culture medium without MPP^+^ applied.

## 3. Experimental Section

### 3.1. Chemicals and Reagents

Human neuroblastoma cell line SH-SY5Y were obtained from Xuanwu Hospital (Beijing, China) as a gift; human glioblastoma multiform cell line U87 and T-cell leukemia Jurkat cells were purchased from Peking Union Medical College (Beijing, China); caspase3/7 (Lot No: 0000054568) and caspase8 (Lot No: 0000052766) kit purchased from Promega (Madison, WI, USA); annexin V-PI flow cytometry analysis kit purchased from Keyueda (Beijing, China; Cat No: KTK101-100); cell-cycle flow cytometry analysis kit purchased from Keyueda (Cat No: KTK102-100); MPP^+^ purchased from Sigma (St. Louis, MO, USA; Cat No: D048-1G); MTT purchased from Genview (Houston, TX, USA; Cat No: JT343), MTS purchased from Promega (Madison, WI, USA, Cat No: G111B), phenazinemethosulfate (PMS) purchased from Sigma (St. Louis, MO, USA; Cat No: P9625); DMSO purchased from Sigma (Cat No: D8428), imobilon^®^-P Transfer Membrane (Lot No: K2KA7282EK) purchased from Millipore (Billerica, MA, USA); Antibodies for β-actin (Billerica MA, USA, Lot No: 3700S), CyclinB1 (Beverly, MA, USA, Lot No: P14635), CDC2 (Beverly, MA, USA, Lot No: 9112S), p-Cdc2 (Beverly, MA, USA, Lot No: 2543S) purchased from Cell Signaling Technology.

### 3.2. Cell Culture

SH-SY5Y cells were maintained in Dulbecco’s Modified Eagle’s Medium (Gibco^®^, São Paulo, Brazil); U87 cells were maintained in Dulbecco’s Modified Medium (Hyclone™, Basingstoke, UK) supplemented with 1× minimum essential medium nonessential amino acids (MEM NEAA); Jurkat cells were maintained in RPMI medium 1640 basic (Gibco^®^). All media were supplemented with 10% fetal bovine serum (Gibco^®^) and penicillin–streptomycin. The cells were cultured at 37 °C in a 5% CO_2_ humidified incubator.

### 3.3. Cell Co-Culture

SH-SY5Y cells were co-cultured with U87 cells as a ratio of 1:1 in completed Dulbecco’s Modified Eagle’s Medium (Gibco^®^) supplemented with 10% fetal serum (Gibco^®^) and penicillin–streptomycin in 100 mm plates at 37 °C in a humidified incubator with 5% CO_2_ for 8 h for adhering, then 100 or 500 µM MPP^+^ for 24 h were added, followed by conditioned medium to cultivate Jurkat cells at 37 °C for another 24 h to derive the medium from U87 or SH-SY5Y cells treated with or without MPP^+^ as control.

### 3.4. Caspase3/8 Activity Assays

Caspase3 activity was measured by Promega caspase3/7 activity kit according to the instruction. Briefly, Jurkat cells were harvested and resuspended with Dulbecco’s Modified Eagle’s Medium (Gibco^®^) to regulate cell concentration to 1 × 10^5^ cells/mL, and then 1 × 10^4^ cells were given to the followed treatment. The fluorescence of caspase3 activity was analyzed by Promega Subscription Fluorescence Detector (GLOMAX 20/20 LUMINOMETER, Madison, WI, USA). Meanwhile, the living cell numbers were also used to estimate cell viability and diameter by Millpore Scepter (Handheld Automated Cell Counter, Millipore, Billerica, MA, USA) [18].

### 3.5. Cell Viability Assay

The viability of SH-SY5Y, U87 cells treating with different concentrations (50 to 1000 μM) of MPP^+^ were evaluated by MTT assay. The viability of Jurkat cells were evaluated by MTS assay in the presence of phenazinemethosulfate (PMS). All experiments were repeated at least three times and finally the cell viability was calculated as follows: cell viability (%) = OD (the experimental group)/OD (control) × 100%.

### 3.6. Western Blot

Jurkat cells were harvested and lysed at 24 h *post* incubation, and total proteins were collected and separated in sodium dodecyl sulfate-polyacrylamide gel electrophoresis (SDS-PAGE) for further immunological assay. The primary antibodies of β-actin (1:4000), CDC2 (1:1000) and CyclinB1 (1:1000), horseradish peroxidase-conjugated goat anti-mouse IgG (1:4000) for β-actin, and goat anti-rabbit (1:4000) for CDC2 and CyclinB1 were used to detect positive signal. And then specific protein bands were visualized with chemiluminescence reagents (Thermo, Madison, WI, USA) by Bio-lab Molecular Imaginer^®^ChemiDoc™ XRS+. The densitometry of the bands was analyzed by Bio-Rad imaging-lad 4.0 software (Bio-Rad, Richmond, CA, USA).

### 3.7. Flow Cytometry Analysis

Jurkat cells were collected at 24 h after cultivation with the conditioned medium, and then some of them were stained with PI for cell-cycle analysis. The Hochest33342 and PI double staining for necrosis analysis in flow cytometry (BECKMAN Cytomics FC 500, Beckman, Palo Alto, CA, USA) were performed on the other part of the cells; Hochest33342 UV channel and PI red fluorescence channel (detection PE-Cy5orPerCP-Cy5.5channels) were detected. Data are collected from three independent experiments.

### 3.8. Transmission Electron Microscopy (TEM) Assay

Jurkat cells (about 5 × 10^6^ cells) were collected at 24 h after cultivation with the conditioned medium, and according to the standard protocol (such as fixation, dehydration, embedding), finally ultrathin transverse sections (70 nm) were sliced and given double staining with plumbum and uranium. The samples were observed by transmission electronmicroscopy (TEM) (Japan, Hitachi, H-7650B) in the Biomedical Testing Center of Tsinghua University (Beijing, China).

### 3.9. Statistical Analysis

Student’s *t*-test or ANOVA (analysis of variance) assay were conducted to evaluate the difference between the experiment and control groups. Significance was set at *p* < 0.05.

## 4. Conclusions

Glial cell activation plays an important role in the pathogenesis of neuron degeneration disease. The neuron–glial co-culture system in which these two cells physically came in contact with each other or the semi-permeable transwell insert system was widely used to study neuron–glial interaction in Parkinson’s disease [17], but the cell co-culture system was rarely used in immune function study. As 100 µM MPP^+^ damaged 19.9% SH-SY5Y cells and 500 µM MPP^+^ reached semi-lethal levels of SH-SY5Y cells, at the same time both of these two doses could keep more than 93.4% Jurkat cells alive (Figure 1). These two concentrations of MPP^+^ could be used with both slightly and severely damaged neuroblastoma cells to learn about T-cell function in PD in a co-culture cell model. Here, we successfully used a three-cell co-culture simple model, including neuroblastoma cells SH-SY5Y, glioma cells U87 and T-cell leukemia Jurkat, to study the peripheral immune effect in PD.

Neurontoxin 1-methyl-4-phenyl-1,2,3,6-tetrahydropyridine (MPTP) is one of the environmental factors in PD [19]. MPTP-mediated toxicity is induced by 1-methyl-4-phenyl-2,3-dihydropyridiumion (MPP^+^), which was converted by monoamine oxidase B in astrocytes [20]. Recently, many studies showed that damaged substantianigra striatum regions had T, B lymphocyte cell infiltration in PD, and damaged regions had high levels of inflammatory factor, tumour necrosis factor α (TNF-α), interleukin 1β (IL-1β), IL-1, IL-2, IL-4, IL-6 and some chemokines [21,22,23,24]. Further studies found the peripheral T-cells pool had been changed, specifically that CD4^+^ T cells had been decreased in PD [12,14,25]. In our study, we observed the similar change of peripheral T cells by the co-culture cell model (Figure 2). However, it is interesting that our data implies that damaged dopamine neurons without glial cells cannot induce a decrease in T-cell population, while the co-culture system of damaged neuronal and glial cells can inhibit proliferation of Jurkat cells, which may contribute to reduced peripheral immune function in Parkinson’s disease. The mechanism of T-cell inhibition induced by damaged dopamine neurons and glial cells should be further investigated.

Cell-cycle checkpoint could inhibit cell growth and then reduce cell number. There were at least two cell-cycle checkpoints in cellular response, G1/S and G2/M, which allowed DNA to repair before DNA duplication and mitosis occurred, respectively [26]. CDC2 regulates mitosis and binds to Cyclin B to form the mitosis-promoting factor (MPF) [27]. MPF was regulated by the phosphorylation/dephosphorylating of CDC2 and the accumulation of Cyclin B [28]. In our study, it was shown that more than 10% of Jurkat cells in 100 µM MPP^+^ applied co-culture medium were arrested at G2/M via decreased phosphorylation of CDC2 and dissociation with Cyclin B1 (Figure 3b,c). It was possible that G2/M cell-cycle checkpoint delayed cell-cycle progression to make more time for DNA repairing, thus inhibiting proliferation of Jurkat cells.

In response to many insults, apoptosis and necrosis became a continuum pathway. Apoptosis happened at a lower dose and necrosis at a higher dose [29]. In this paper, the lower dose of 100 µM MPP^+^-applied co-culture medium made Jurkat cell G2/M cell-cycle checkpoint and no apoptosis, while the higher 500 µM dose induced necrosis of Jurkat cells with a larger diameter and broken membrane (Figure 4). One hundred micro moles per liter MPP^+^-applied co-culture medium caused Jurkat cells to remain in the G2/M cell-cycle checkpoint, while 500 µM MPP^+^ damaged Jurkat cell membrane and engendered necrosis. These results demonstrated that as the concentration of MPP^+^ increased, damaged SH-SY5Y cells inhibited proliferation of Jurkat cells from the G2/M cell-cycle checkpoint to necrosis. The results indicated that one of the factors in the co-culture medium induced by MPP^+^ injured the Jurkat more seriously and was more damaging than the SH-SY5Y cells. We still do not know what factor it was, and we need to study further to find the answer.

Some studies reported immunodeficiency mice which lack T-cells were resistant to MPTP [30], and also found in MPTP-treated T cell receptor beta (TCR β^−^), CD4^−^ and CD8^−^-deficient mice, transferred CD4^+^ T cells accelerated nigral degeneration, whereas CD8^+^ T cells did not [10]. All of our results also indicated neurotoxin MPP^+^-damaged dopamine neurons could suppress immune function of T cells, and this could be one reason behind the aggravation of PD progression.

In conclusion, MPP^+^-damaged SH-SY5Y cells could inhibit proliferation of Jurkat cells along with assistant U87 cells. We further found that a mechanism of this inhibition was 100 µM MPP^+^ applied co-culture medium causing Jurkat cells to stay in the G2/M cell-cycle checkpoint, while 500 µM MPP^+^ damaged Jurkat cell membranes and caused necrosis in cells.

## Abbreviations

PD, Parkinson’s disease; MPTP, 1-methyl-4-phenyl-1,2,3,6-tetrahy-dropyridine; MPP^+^, 1-methyl-4-phenyl-2,3-dihydropyridiumion; SNpc, Substantianigra pars compacta; PI, Propidium iodide; MPF, Mitosis-promoting factor.
